# i-motifs: DNA structures with regulatory functions and therapeutic potential

**DOI:** 10.31744/einstein_journal/2026RW1900

**Published:** 2026-06-23

**Authors:** Murilo Porfírio de Aguiar, Eleni Solange de Brito Gomes, Jéssica Ferreira Vieira

**Affiliations:** 1 Barretos Cancer Hospital Molecular Oncology Research Center Barretos SP Brazil Molecular Oncology Research Center, Barretos Cancer Hospital, Barretos, SP, Brazil.; 2 Universidade Federal do Triângulo Mineiro Human Genetics Laboratory Uberaba MG Brazil Human Genetics Laboratory, Universidade Federal do Triângulo Mineiro, Uberaba, MG, Brazil.; 3 Universidade Federal do Triângulo Mineiro Programa de Pós-Graduação em Ciências da Saúde Uberaba MG Brazil Programa de Pós-Graduação em Ciências da Saúde, Universidade Federal do Triângulo Mineiro, Uberaba, MG, Brazil.

**Keywords:** Nucleic acid conformation, Molecular targeted therapy, Gene expression regulation, Oncogenes, Promoter regions, genetics

## Abstract

Non-B DNA structures represent alternative conformations to the canonical double helix, characterized by unconventional base pairing that diverge from the Watson-Crick model. Among these, the i-motifs, formed in cytosine-rich regions, has attracted considerable attention due to its potential regulatory roles. This review examines the molecular mechanisms underlying the formation and stability of i-motifs, which rely on hydrogen bonding between protonated cytosines. These structures can form under slightly acidic and even neutral pH conditions, depending on the DNA sequence context. Their diverse topologies and capacity to interact with specific proteins and ligands suggest that i-motifs play important roles in the regulation of gene expression, particularly within promoters of oncogenes, as well as in telomeric and centromeric regions. Understanding the behavior and biological relevance of i-motifs may deepen our insight into genomic regulation and reveal novel therapeutic opportunities. Targeting i-motif structures therefore represents a promising strategy for the treatment of cancer and other complex diseases.

## INTRODUCTION

The discovery of the DNA structure by Watson et al. in 1953 marked the beginning of a revolution in molecular biology. The double helix has since become the canonical model of genetic material, representing the mechanism by which biological information is stored and transmitted across generations. In this structure, the nitrogenous bases adenine and thymine are connected by two hydrogen bonds, whereas guanine and cytosine are linked by three.^(1)^ However, the double helix does not encompass the full structural diversity of DNA. More recent studies have identified alternative conformations, known as non-B DNA structures, among which the i-motif has attracted significant attention.^(2)^

**Figure 1:**
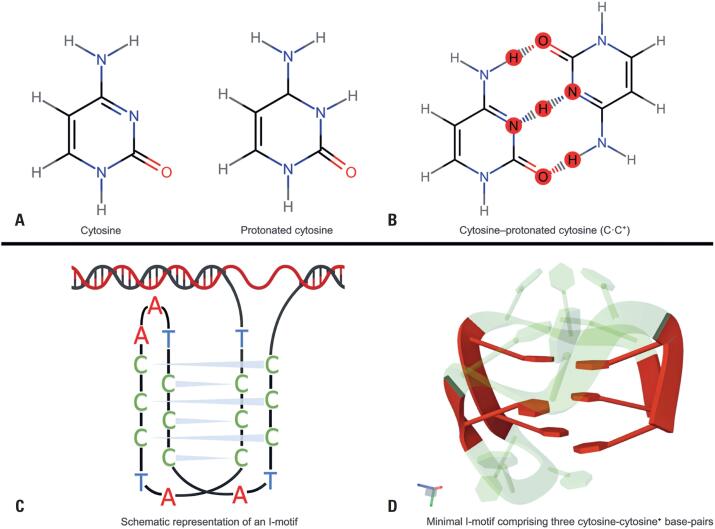
Protonated cytosine and C·C^+^ base pairing as the molecular basis of i-motif formation. (A) Chemical structures of neutral cytosine and protonated cytosine (C^+^), in which the additional proton is located at the N3 position. (B) Hemiprotonated cytosine–cytosine (C·C^+^) base pair. In this schematic, three hydrogen-bonding interactions are shown: one between the protonated N3–H^+^ and the N3 of the opposing base, and two involving the amino group (–NH_2_) and the carbonyl oxygen (C=O). Although three interactions are shown here for completeness, the formation of two hydrogen bonds (N3–H^+^···N3 and NH_2_–H···O=C) is generally sufficient and most commonly described in the literature as stabilizing the C·C^+^ base pair (adapted from Benabou et al., 2014).^(11)^ (C) Schematic representation of the i-motif fold, highlighting the intercalated C·C^+^ base pairs that stabilize the structure. (D) Three-dimensional i-motif structure comprising three C·C^+^ base pairs (PDB ID: 8PMB), illustrating the characteristic tetrahelical fold (reported by Ghezzo et al., 2023).^(12)^ The three-dimensional structure of the i-motif is relatively compact, consisting of four intercalated DNA strands arranged in an antiparallel orientation, with cytosine bases stacking to stabilize the structure. Each layer of hemiprotonated cytosines stacks upon the next, promoting additional interactions that maintain structural integrity. However, the i-motif can adopt different topologies - i.e., distinct three-dimensional patterns - depending on the nucleotide sequence (Figure 2). These base-stacking interactions are similar to those observed in other non-B structures, such as G-quadruplexes; however, i-motif stability specifically depends on cytosine protonation.^(13)^ Van der Waals forces between stacked cytosine layers also contribute significantly to the structural stability

The i-motif is notable for its unconventional base-pairing pattern: instead of pairing with guanine, as is typical for cytosine, two cytosines interact directly, forming a hemiprotonated C·C^+^ base pair under specific conditions, typically at slightly acidic pH (Figure 1A-B).^(3)^ This seemingly counter-intuitive phenomenon is increasingly recognized as part of a more versatile structural repertoire of DNA than previously appreciated. Interestingly, the term "i-motif" was used in scientific contexts even before its identification in human DNA, suggesting that this configuration may have implications beyond the human genome, including in fields such as virology.^(4)^

**Figure 2:**
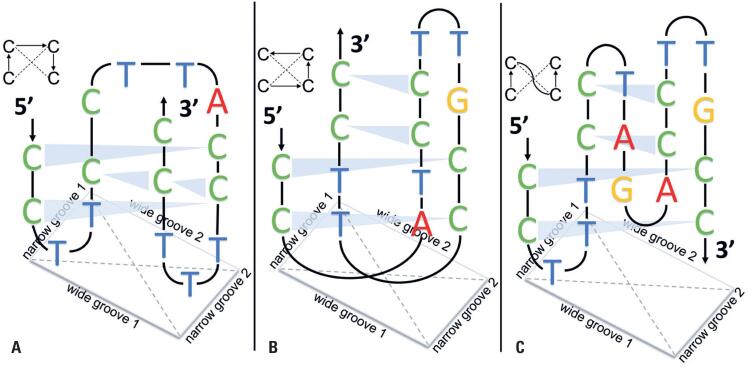
Schematic representation of different i-motif topologies, illustrating how variations in nucleotide sequence influence the spatial arrangement of four antiparallel DNA strands. (A) Basic organization of the i-motif, with stacked hemiprotonated cytosines base pairs (C·C^+^) forming the layers. The 5’ and 3’ ends are indicated, along with the "wide" and "narrow" clefts. (B) and (C) Alternative topologies, in which other bases (G, A, and T) are present in loops or at the termini, altering the overall conformation. Black arrows and curves illustrate strand connectivity, while shaded regions represent base stacking, contributing to structural stability. Adapted from Han et al., 1998^(10)^

The pH conditions that favor i-motif formation, ranging from slightly acidic to near-neutral, are not uncommon in animal cells.^(5)^ This helps explain why its presence has been confirmed not only in humans but also in other mammals and even bacteria. ^(6,7)^ Techniques such as nuclear magnetic resonance, UV spectroscopy, and gel chromatography have been used to investigate its properties, revealing both the conditions that stabilize this structure and its potential biological functions.^(^ Although the i-motif is currently one of the most extensively studied non-B DNA structures, it is not the only documented structural variation of DNA. G-quadruplexes, hairpins, cruciforms, and triplex structures are additional examples of non-B conformations that challenge traditional views of DNA's structural rigidity. The ability of DNA to adopt such diverse conformations not only underscores its complexity but also suggests that these variations may play functional roles that remain to be fully elucidated.

In recent years, - a growing body of research has focused on understanding how i-motifs may influence pathological processes, including cancer development. Although studies are still at an early stage, the possibilities are intriguing: these structures may be involved in gene regulatory mechanisms or in alterations that promote disease progression.^(8,9)^ This perspective, while challenging, points to a fertile area of investigation that may redefine our understanding of the genome. Given the evidence presented, this narrative review aims to contextualize and integrate studies on non-B DNA structures, with a particular emphasis on i-motifs. This work synthesizes both theoretical and experimental contributions, highlighting how these structural variations and the molecular mechanisms governing them influence gene expression and are associated with pathological processes, such as cancer development. Additionally, it provides a historical overview of the discovery of these structures and traces the evolution of knowledge over time. By offering a comprehensive and up-to-date perspective on the subject, this review aims to promote innovative therapeutic approaches and stimulate further advances in the field of molecular biology.

### Non-B structures

The i-motif is a secondary DNA structure that forms in cytosine-rich regions, typically under slightly acidic pH conditions. However, evidence suggests that specific sequences, such as d(5mCCT3CCT3ACCT3CC), can adopt the i-motif conformation even at neutral pH.^(10)^ Unlike the conventional B-DNA helix, which relies on Watson-Crick hydrogen bonding between nitrogenous bases, the i-motif depends on unconventional interactions involving protonated cytosines. These interactions occur between two cytosines, one of which must be protonated (i.e., acquire an H^+^) to enable bond formation. The C^+^–C base pair is stabilized by hydrogen bonds: one between the amino group (NH^2^) of one cytosine and the carbonyl group (C=O) of the other, and another between the protonated N3–H^+^ and the N3 of the opposing base (Figure 1B).^(11)^ i-motifs are commonly illustrated in figure 1, where a single DNA strand folds back on itself, forming the characteristic structure distinct from the canonical B-DNA conformation. The three-dimensional structure of the i-motif is highly compact, consisting of four intercalated strands arranged in an antiparallel orientation, with cytosine bases stacking to stabilize the structure (Figure 1D).^(12)^

**Figure 3:**
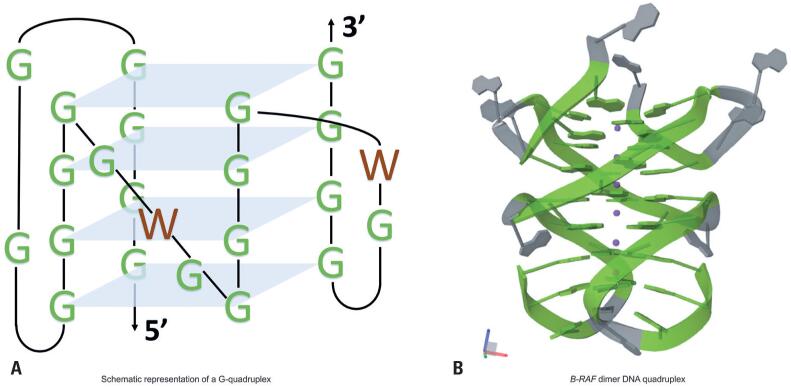
G-quadruplex DNA. (A) Guanine bases are stabilized by Hoogsteen hydrogen bonding; the letter "W" marks positions that can engage in canonical Watson–Crick base pairing. Adapted from Choi & Majima, 2011.^(2)^ (B) Atomic model of the human *BRAF* promoter G-quadruplex (PDB 4H29; X-ray), which adopts an intertwined dimeric arrangement. Guanine residues are shown in green and form the G-quadruplex; while other nucleotides are shown in gray. Potassium ions in the central channel are depicted as purple spheres (structure from Wei et al., 2013)^(14)^

G-quadruplexes (G4s) are four-stranded DNA structures that form in guanine-rich regions.^(14)^ These structures are stabilized by Hoogsteen hydrogen bonding, which differs from canonical Watson-Crick base pairing. Each G4 consists of four guanine bases arranged in a planar configuration, forming a guanine tetrad stabilized by monovalent cations such as potassium (K^+^) or sodium (Na^+^), which are positioned between the tetrads to enhance stability.^(15)^ Depending on sequence length and strand orientation, G4s can adopt parallel, antiparallel, or hybrid conformations. Specific G4 topologies have been associated with particular genomic regions and pathological features, such as in the *PDGFA* gene, which has been linked to metastatic potential (Figure 3).^(16)^

**Figure 4:**
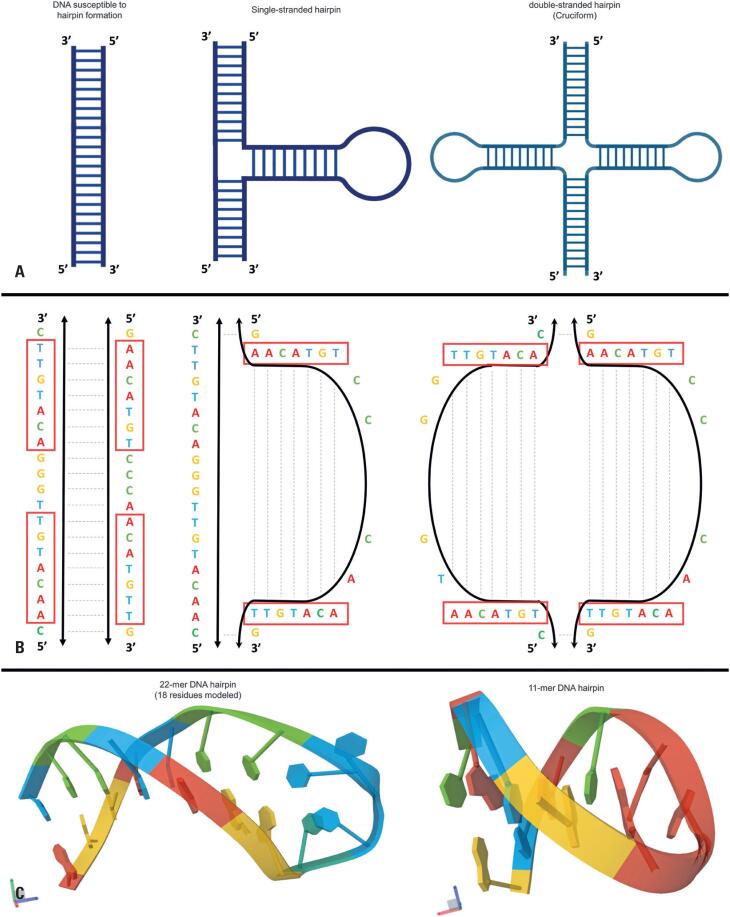
DNA hairpins and cruciforms. (A) Schematic overview. Left: a duplex segment containing an inverted repeat sequence (a short palindromic motif) that is susceptible to hairpin extrusion. Middle: a single-stranded hairpin formed by intramolecular base pairing. Right: a cruciform structure formed when both strands of the inverted repeat fold out to generate two opposing hairpins. (B) Sequence-level view of hairpin formation. Palindromic segments that pair to form the stem are boxed; dashed lines denote Watson–Crick base pairs; arrows indicate the 5′→3′ orientation; adapted from Bansal et al., 2022.^(17)^ (C) Representative three-dimensional hairpin structures from the RCSB Protein Data Bank. Left: an 18-nt (18-nucleotide) hairpin (PDB ID: 1QE7, solution NMR; Ghosh et al., 1999).^(18)^ Right: an 11-nt hairpin (PDB ID 1BJH, solution NMR; Chou et al., 1996).^(19)^ An atomic-resolution three-dimensional cruciform structure is not shown because, to our knowledge, such structures have not been deposited in the PDB as stable, isolated entries. Cruciform formation depends on DNA topology and negative supercoiling and is often transient in short oligonucleotides, making it difficult to capture using NMR or crystallography

Hairpins and cruciforms are structures formed when a single DNA or RNA strand folds back on itself, creating a paired "stem" of complementary bases and an unpaired "loop." These structures commonly arise in inverted repeat sequences, where intramolecular base pairing can occur. When two such hairpin structures form on opposite strands of DNA, they can give rise to a more complex structure known as a cruciform. These structures may interfere with replication and transcription by acting as physical barriers that stall the enzymes involved in these processes. Regions of negative supercoiling favor cruciform formation and are often associated with palindromic repeat sequences (Figure 4).^(17^

**Figure 5:**
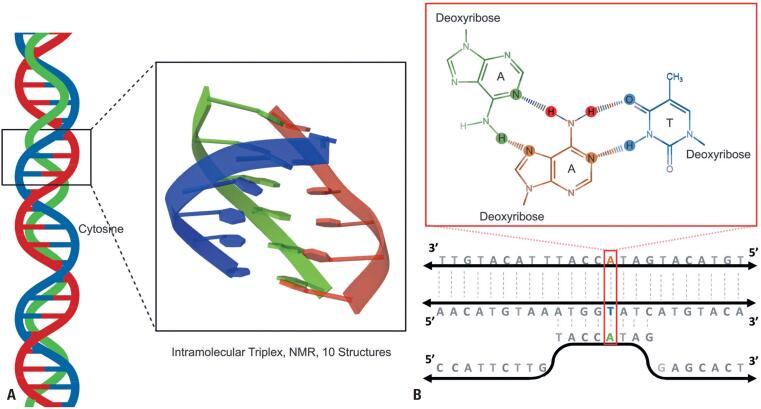
DNA triple helix (triplex) structures. A) Three-dimensional view of an intramolecular DNA triplex solved by solution-state nuclear magnetic resonance (PDB ID: 1BCB). The structure was deposited by Asensio et al. (1998) and classified as DNA without mutations.^(20)^ This triple helical arrangement illustrates the association of a third strand with the canonical duplex, stabilized by Hoogsteen interactions. B) Simplified schematic (adapted from Brazda et al., 2020; and Holder et al., 2015) illustrating the principle of triplex formation.^(22,23)^ The canonical duplex is stabilized by Watson–Crick base pairs, while the third strand binds in the major groove via Hoogsteen hydrogen bonds. The chemical structure shown in green corresponds to adenine from the third strand, forming a representative T·A·T triad with a Watson–Crick A·T base pair. Such alternative base-pairing interactions enable an additional strand to associate with duplex DNA through Hoogsteen hydrogen bonding, generating a triple-helical structure capable of modulating essential processes such as replication and transcription

Triplex structures, or triple helices, form when a third DNA strand binds to the canonical double helix. This third strand may originate from a separate DNA molecule or from a different region of the same molecule, folding back to interact with the double helix (Figure 5).^(20)^ The third strand binds to the double helix through Hoogsteen or reverse Hoogsteen hydrogen bonding, which differs from canonical Watson-Crick base pairing.^(21)^ In Watson-Crick base pairing, adenine (A) pairs with thymine (T) via two hydrogen bonds, while guanine (G) pairs with cytosine (C) via three hydrogen bonds. In Hoogsteen bonding, the base-pairing geometry is altered, allowing a third base to interact with purines (A or G) in one strand of the double helix. Purines, due to their larger size and two-ring structure (compared to the single-ring structure of pyrimidines), are more accessible for these additional interactions.^(2,22,23)^ This three-stranded structure can interfere with critical processes such as DNA replication and transcription

Triplex structures may be recognized as DNA damage by repair proteins such as ERCC1-XPF and XPG. These proteins, which typically detect and repair genuine DNA lesions, may identify triple helices as damaged and attempt to process them. During this process, they may inadvertently introduce DNA breaks, thereby increasing genomic instability and the risk of mutations and chromosomal rearrangements.^(21)^

### Biological functions of the i-motif structure

The i-motif is a secondary DNA structure characterized as a four-stranded non-covalent complex that forms in cytosine-rich sequences, typically under slightly acidic pH conditions.^(2)^ Unlike the canonical DNA double helix (B-DNA), i-motifs have been less extensively studied than other non-B structures, such as G-quadruplexes. However, interest in these structures has grown significantly in recent years, driven by their potential regulatory roles in biological processes, particularly in the modulation of gene expression. Their biological functions are closely linked to their ability to interact with various molecules, including proteins, small ligands, and epigenetic modifications.^(24,25)^

I-motifs are proposed to act as molecular "on-off" switches for gene expression, influencing essential cellular processes such as replication, transcription, and translation.^(26,27)^ These structures are found in several regulatory regions of the human genome, including promoters, telomeres, centromeres, and ribosomal DNA regions.^(24,26)^ Their presence in gene promoters, for example, underscores their structural diversity, including variations in topology, loop length, and transition pH. This versatility makes them promising targets for investigating their biological functions and for the development of small-molecule-based therapies.^(26,27)^

The interaction of i-motifs with other molecules is crucial for their biological functions, although the precise mechanisms underlying gene expression regulation are not yet fully understood. Evidence suggests that i-motifs interact with DNA-binding proteins, serving as recognition sites for transcription factors or repressors.^(28-30)^ These interactions may modulate gene expression by either facilitating or hindering the binding of regulatory proteins. In some cases, i-motifs physically impede transcription factor binding, thereby repressing gene expression.^(26)^

On the other hand, specific ligands may induce conformational changes in the i-motif, thereby increasing its affinity for regulatory proteins and leading to transcriptional activation and enhanced gene expression.^(26,27)^ The interaction of i-motifs with proteins such as hnRNP LL and hnRNP K has been extensively studied. For instance, hnRNP LL interacts with i-motifs, promoting their unfolding and the formation of a stable single-stranded structure, which facilitates transcriptional activation.^(31,32)^ Similarly, hnRNP K has been shown to unfold the i-motif in the *KRAS* gene region, thereby positively regulating its expression. Additionally, i-motifs formation may compete with other DNA structures, such as G-quadruplexes, which are also involved in gene regulation.^(33)^ The transition between these conformations may be modulated by environmental factors, such as pH and ionic strength, providing a dynamic mechanism for the control of gene expression.^(15,26)^ The influence of ligands on i-motif-mediated gene expression is complex and multifaceted. Ligands may modulate i-motif stability, either stabilizing or destabilizing the structure. In some cases, stabilization may prevent transcription factor binding, leading to gene repression, or impede RNA polymerase progression, thereby inhibiting transcription.^(24,34)^ A notable example is the natural compound hyoscine butylbromide, which specifically binds to the G-quadruplex within the *C-MYB* gene.^(35)^ Furthermore, ligands may influence epigenetic processes, such as DNA methylation, thereby indirectly affecting gene expression.^(36-38)^ Compounds such as IMC-48 and IMC-76 have been shown to modulate gene expression by interacting with i-motif-forming sequences in the *BCL2* promoter, exerting opposite effects on *BCL2* regulation.^(26,39)^ Another example is the stabilization of telomeric i-motifs by carboxylated single-walled carbon nanotubes (SWCNTs), which inhibit telomerase activity and interfere with telomere functions in cancer cells.^(40)^

The stability of i-motifs under physiological conditions is a critical factor for their biological relevance. Although i-motifs were initially thought to be stable only at acidic pH, recent studies have that some can form at neutral pH, particularly in sequences containing at least five consecutive cytosines.^(40)^ In addition to pH, factors such as low temperature, molecular crowding,^(13)^ negative supercoiling,^(28-30)^ and the presence of silver (I) cations also influence i-motif stability.^(29)^ A notable example is the i-motif-forming sequence in the promoter region of *HIF-1*α, which exhibits stability at near-neutral pH.^(5)^

In the therapeutic context, i-motifs are frequently found in oncogene promoter regions and may be considered complementary to G-quadruplexes.^(26)^ Given the role of G-quadruplexes in telomerase inhibition, transcriptional regulation, and replication, it is plausible that i-motifs also exert regulatory functions, either as primary elements or by contributing to the stabilization of G-quadruplexes relative to the double helix. This versatility makes i-motifs promising targets for chemical intervention, particularly in contexts in which they may directly modulate transcription.^(40)^ A relevant example is the stabilization of telomeric i-motifs by carboxylated single-walled carbon nanotubes, which inhibit telomerase activity and interfere with telomere function in cancer cells.^(41)^

In conclusion, i-motifs are non-canonical DNA structures that appear to play significant roles in biological processes, particularly in the regulation of gene expression. Although research on i-motifs is still at an early stage, current evidence highlights their potential as therapeutic targets for a variety of diseases. Further studies are needed to fully elucidate their biological functions and to develop small molecules capable of specifically modulating these structures for therapeutic purposes.

### Implications of i-motif structures in carcinogenesis

Cytosines and guanine-rich regions are frequently found in oncogene promoters, centromeres, and telomeres. These regions have the potential to form specific non-canonical DNA structures, such as G-quadruplexes and i-motifs.^(28,42)^

Since i-motifs are formed by cytosines, nucleotides that represent the primary targets of epigenetic modifications in the human genome, they serve as potential sites for epigenetic regulation. These modifications can directly influence crucial biological processes^(38)^ as changes at the 5-position of cytosines within CpG dinucleotides affect the thermal stability of non-canonical structures, particularly i-motifs.^(25,43)^

In promoter regions, i-motifs may act as molecular regulators, modulating gene expression and contributing to genome integrity.^(36)^ Their abundance increases during the G1/S phase of the cell cycle, a period marked by intense transcriptional activity. This association highlights a possible link between i-motifs and cancer development, as these structures are thought to function as recognition sites for proteins involved in transcriptional activation.^(44)^ Furthermore, i-motifs have been identified in the promoters of several cancer-related genes, including *HRAS,*^(32)^ *KRAS,*^(45)^ *VEGFA,*^(46)^ *RET,*^(47)^ *BCL2,*^(48)^ *RAD17,*^(49)^ *MYC,*^(50)^ *MYB,*^(51)^ *KIT,*^(52)^ *PDGFR-B,*^(53)^ *HIF-1A,*^(54)^ *RB1,*^(55)^ and *TERT* (Table 1).^(56)^

**Table 1:** Relationship between cancer-related genes, their functions, and i-motif-mediated transcriptional modulation: summary of gene functions and i-motif-associated transcriptional regulation, highlighting evidence reported in the scientific literature

Cancer-related genes	Function	i-motif-mediated transcriptional modulation
*HRAS* / *KRAS*	This gene family is involved in cellular proliferation, differentiation, and survival^(57)^ and is among the most frequently mutated in cancer^(9)^	i-motif structures facilitate gene transcription through stable binding of the hnRNP A1 protein^(32)^ and through positive regulation mediated by hnRNP K ^(45)^
*VEGFA*	A diffusible endothelial cell-specific mitogen involved in the regulation of physiological and pathological angiogenesis, cell survival, tumor growth, and metastasis^(58)^	Transcriptional activation is mediated by binding of the hnRNP K protein to the partially unfolded i-motif structure^(46)^
*RET*	Involved in tissue development, embryogenesis, immune surveillance, and metabolic regulation^(59)^	To the best of our knowledge, no evidence has been reported in the literature
*BCL2*	Regulator of the mitochondrial (intrinsic) apoptotic pathway, promoting cell survival by inhibiting cell death^(60)^	Transcriptional activation is mediated by the hnRNP LL protein^(48)^
*RAD17*	Responsible for DNA damage checkpoint control^(61)^	To the best of our knowledge, no evidence has been reported in the literature
*MYC*	Regulates cellular proliferation, differentiation, and apoptosis^(62)^	The single-stranded CT-element – facilitated by dynamic structures such as the i-motif – enables hnRNP K binding, which acts as a transcriptional activator and leads to increased gene expression^(50,63)^
*MYB*	Regulates cellular proliferation and differentiation in the hematopoietic and gastrointestinal systems; its overexpression induces cancerous lesions^(64)^	To the best of our knowledge, no evidence has been reported in the literature.
*KIT*	Regulates the proliferation and differentiation of hematopoietic stem cells and contributes to maintenance of the immune system^(65,66)^	Repression of gene transcription and translation following i-motif stabilization by the bisacridine derivative B05^(52)^
*PDGFR-B*	Promotes tumor growth through autocrine stimulation of malignant cells^(67)^	Transcriptional activation is favored by the binding of activator proteins to the i-motif located in the gene promoter^(53)^
*HIF-1A*	Related to metabolic adaptation, erythropoiesis, angiogenesis, cell growth and differentiation, survival, and apoptosis^(68)^	To the best of our knowledge, no evidence has been reported in the literature
*RB1*	Tumor suppressor that restricts the expression of genes required for cell proliferation^(69)^	Destabilization of the i-motif allows *RB1* transcription to increase^(55)^
*TERT*	Responsible for replication of human telomere ends, thereby promoting cell survival^(70)^	The presence of the i-motif does not favor the emergence of TERT activating mutations^(56)^

The presence of i-motif structures in the promoter regions of oncogenes, as mentioned earlier, does not directly alter gene transcription. However, their stability and conformation can be modulated by proteins or small molecules. These interactions may lead to either activation or repression of gene expression, highlighting the dynamic regulatory role of these structures.

Although i-motifs are recognized as regulatory elements, demonstrating their functionality in vivo remains a significant challenge. These structures are highly dynamic and transient, making them difficult to detect under physiological conditions. While early studies suggested that i-motifs were stable only in acidic environments, more recent work has shown that specific sequences, such as those in *HIF-1A* and telomeric regions, fold at near neutral pH.^(13,40)^ However, such stability is context-dependent, and factors such as negative supercoiling, molecular crowding, and protein interactions are crucial for their formation. This complexity has fueled ongoing debate as to whether i-motifs function as regulatory switches or merely represent transient structural intermediates.

Although i-motifs have been identified in numerous genes, as summarized in table 1, the functional relationship between i-motif formation and gene transcription remains unclear for many of them. This highlights a substantial gap in our understanding of their biological significance.

Another limitation lies in the methodological constraints of current detection tools. Traditional biophysical approaches, such as circular dichroism and NMR spectroscopy, are often performed under non-physiological conditions and cannot fully capture the dynamics of i-motif folding in living cells. Although emerging strategies, such as fluorescent probes, i-motif–specific antibodies, and in-cell NMR spectroscopy, have provided compelling evidence of their presence in vivo, these techniques still face challenges related to sensitivity, specificity, and spatial resolution.^(13)^

### i-motifs in centromeres and telomeres: implications for chromosomal stability

Centromeres, the chromosomal regions where kinetochores (multiprotein structures responsible for attaching chromosomes to spindle microtubules) are assembled, are essential for proper chromosome segregation and sister chromatid separation during cell division.^(71,72)^ Alterations in centromeric stability are closely associated with tumor development.^(72)^ Studies have identified i-motif structures in centromeric satellite DNA, and their presence has been positively associated with the structural and functional organization of centromeres.^(73,74)^ Evidence suggests that defects in centromere formation and maintenance may precede tumor cell development, as disruptions in these domains have been frequently observed across various cancer types.^(75,76)^ This suggests a potential protective role of i-motifs in limiting tumor initiation and progression.

Complementarily, telomeres– nucleoprotein complexes located at chromosome ends that prevent inappropriate DNA repair responses, contain guanine- and cytosine-rich sequences capable of adopting G-quadruplex and i-motif conformations.^(15,77)^ Telomere stability is regulated by the enzyme telomerase, whose activity is crucial in carcinogenesis, as telomere maintenance is a common feature of various tumor cell types.^(78)^ The formation of G-quadruplex and i-motif structures at telomeres has been associated with telomerase inhibition, leading to telomere uncapping and subsequent activation of a DNA damage response, which may ultimately result in growth arrest and compromise cancer cell survival.^(41,79)^

### Implications of i-motifs in pathological conditions beyond cancer

I-motif mediated gene regulation presents promising applications in pathological conditions beyond cancer. In hepatic disorders such as non-alcoholic steatohepatitis (NASH), stabilization of the i-motif in the *BCL-2* gene has been explored as an innovative therapeutic strategy. The use of A22, an acridone derivative, demonstrated significant efficacy in stabilizing the i-motif located in the *BCL2* promoter, resulting in increased transcription and reduced apoptosis in hepatocytes. In animal models, this approach concurrently improved metabolic and inflammatory markers, suggesting a systemic impact rarely observed with single-target therapeutic strategies.^(8)^

The investigation into the relationship between i-motifs and other diseases has also expanded to neuropsychiatric conditions. Thorne et al. examined this association in the context of neuropsychiatric disorders. They demonstrated that the *SLC6A4* gene, responsible for encoding the serotonin transporter, contains allelic variants (*VNTR-S* and *VNTR-L*) that modulate transporter expression and influence susceptibility to psychiatric disorders. These variants are enriched in cytosine-guanine-rich regions, suggesting the potential formation of i-motifs. In vitro spectroscopy confirmed the folding of these regions into i-motif structures, indicating that such conformations may also occur in vivo, potentially regulating *SLC6A4 expression.*^(80)^

Another relevant study on i-motifs, this time in the context of viral infections, was conducted by Ruggiero et al. The authors demonstrated that the HIV genome contains a region known as the long terminal repeat (LTR), which is essential for viral replication following integration into the human genome. This region is enriched in cytosine- and guanine- rich sequences capable of forming i-motifs, whose stabilization inhibits LTR transcription and consequently viral replication. Based on this premise, the study investigated the hnRNP K protein as a stabilizer of the i-motif within this region, enhancing its resistance to variations in pH and temperature. Stabilization of i-motif structures in the LTR showed potential to reduce viral replication, representing an innovative strategy for the development of antiretroviral therapies targeting this region.^(81)^

Beyond their role in gene regulation, i-motifs have also been explored as functional components for controlled drug delivery. Hu et al. developed a DNA hydrogel capable of carrying insulin while resisting the acidic environment of the stomach and subsequently releasing the drug in the intestine. The hydrogel's structure, enriched in hemiprotonated cytosine bases that favor i-motif formation, provided stability to the system until it reached the intestinal tract. The results demonstrated the potential of this approach for drug administration in diabetes treatment, ensuring controlled release throughout the gastrointestinal tract.^(82)^

## CONCLUSION

Recent advances have significantly expanded our understanding of i-motif DNA structures, positioning them as dynamic elements with potential regulatory and therapeutic implications. Although traditionally regarded as transient intermediates, studies published over the past five years have shown that certain i-motifs can form and remain stable under near-physiological conditions, particularly in regulatory regions such as promoters, telomeres, and centromeres. These developments have been driven by the advent of high-resolution imaging, real-time detection systems, and structure-specific probes, most notably the use of i-motif, specific antibodies, and in-cell NMR, which have provided unprecedented insight into their dynamics in living cells.

Despite these advances, key questions remain unanswered, including whether i-motifs exert causal regulatory roles or act primarily as structural intermediates, how their formation is orchestrated during the cell cycle, and to what extent they contribute to genome stability and disease pathogenesis. Addressing these gaps will require integrated strategies combining genome editing (e.g., CRISPR/Cas), structure-targeting ligands, and multi-omics approaches to map their biological functions with greater resolution and context specificity.

Future research should also prioritize functional validation in model organisms and leverage single-molecule biophysics to link i-motif folding dynamics to transcriptional control. Such studies will be crucial for determining whether i-motifs are viable therapeutic targets and for translating these findings into innovative interventions.

In conclusion, i-motifs represent structurally unique and biologically significant DNA elements, offering a fertile field for discovery at the interface of structural biology, epigenetics, and translational medicine. A deeper mechanistic understanding will be essential to fully realize their potential as biomarkers and therapeutic targets in cancer and other complex diseases.

## Data Availability

The underlying content is contained within the manuscript.
